# Managing pregnant women with cancer: personal considerations and a review of the literature

**DOI:** 10.3332/ecancer.2011.204

**Published:** 2011-02-14

**Authors:** HA Azim, O Gentilini, M Locatelli, E Ciriello, G Scarfone, FA Peccatori

**Affiliations:** 1 Department of Medical Oncology, Jules Bordet Institute, Brussels, Belgium; 2 Department of Surgery, Division of Senology, European Institute of Oncology, Milan, Italy; 3 Department of Medicine, Division of Medical Oncology, European Institute of Oncology, Milan, Italy; 4 Department of Obstetrics and Gynecology, Fondazione IRCCS Ca’ Granda, Ospedale Maggiore Policlinico, Milan, Italy; 5 Department of Obstetrics and Gynecology, Ospedali Riuniti di Bergamo, Bergamo, Italy; 6 Department of Medicine, Division of Hematology Oncology, European Institute of Oncology, Milan, Italy

## Abstract

Managing cancer during pregnancy is a very critical clinical situation. It is relatively rare but once encountered, it poses several clinical and sometimes social and ethical conflicts as well. Generalizing treatment decisions is very hard and in our opinion, each case should be discussed in a multidisciplinary manner acknowledging patients’ opinion as well to reach a proper decision. In this review we touch on the available evidence on managing cancer patients diagnosed during the course of pregnancy in an attempt to provide some guidance for clinicians dealing with such cases.

## Introduction

The diagnosis of cancer during the course of pregnancy is a challenging clinical situation both for the patient and for the treating physicians. It is estimated that one in every 1000 pregnancies is complicated with cancer. The most common tumours diagnosed during pregnancy are breast and cervical cancer followed by melanoma, leukaemia and lymphoma [1]. Given its relative rarity, evidence remains scarce as it is practically impossible to perform large prospective clinical trials. Thus current knowledge is derived from small phase 2 studies, retrospective analyses and systematic literature reviews. Another critical issue is the potential conflict between maternal and foetal well-being: this could result in treating pregnant women for fear of foetal toxicity, or offering therapy that could result in foetal morbidity and/or mortality. In some instances, therapeutic abortion is required, particularly when highly proliferative malignancies are diagnosed in the first trimester of pregnancy. Before the 14th week of pregnancy, chemotherapy is contraindicated for the high risk of foetal malformations and thus there is an urgent need to customize treatment strategies [2]. Although there are some general guidelines that can be applied for all tumour types (Table 1), each type has specific features that should be considered. In this review, we will consider the available evidence for managing pregnant women with cancer in order to provide some guidance for physicians dealing with these patients.

## Breast cancer

Breast cancer is the most common tumour diagnosed during pregnancy with an estimated 10,000 cases diagnosed every year worldwide. Pregnant breast cancer patients are commonly diagnosed with advanced stage of the disease, which is mainly due to diagnostic delay [3]. Local intervention (surgery and radiotherapy) in addition to systemic therapy such as chemotherapy, hormonal therapy and targeted agents have all been described in managing pregnant breast cancer patients.

Available evidence strongly suggests that surgery can be performed safely at any time throughout the course of the pregnancy. The decision to proceed with conservative breast surgery or mastectomy should be decided according to the clinical situation of each patient. There are no strong evidences to support mastectomy for the risk of delaying radiation therapy. In fact, recent guidelines suggest that conservative surgery can be performed during any trimester [4]. Sentinel lymph node (SLN) biopsy has been discouraged by some, despite the absence of evidence showing detrimental effects. This was based on concerns that the foetus could be exposed to the radiolabelled tracer that could potentially result in foetal abnormalities. However, an earlier dosimetry study followed by a recent prospective study on 12 pregnant breast cancer patients by the same group showed no congenital abnormalities and no evidence of axillary recurrence at a median follow-up of 32 months [5,6]. Thus SLN could be considered in selected patients and in centres with experience in carrying out this technique.

Several publications have addressed the feasibility and safety of chemotherapy during pregnancy. Anthracyclines are the most commonly used agents and have demonstrated an apparent safety when administered starting at the second trimester [7–9]. Only two prospectively treated series were described in the adjuvant (neo) setting [10,11]. The first involved 57 patients, who were treated with FAC (5-flourouracil, doxorubicin, and cyclophosphamide) with doxorubicin given as a continuous infusion for 96 hours [10]. The second was published later by our group and involved 20 patients who were treated with weekly epirubicin 35 mg/m^2^ [11]. Both regimens were well tolerated with no apparent increase in the risk of pregnancy complications or foetal congenital abnormalities. Hence, both options remain valid for treating breast cancer patients diagnosed during pregnancy. From a feasibility perspective, the continuous infusion of doxorubicin is not widely adopted and thus might not be convenient during pregnancy. A potential advantage of the later regimen is that the weekly fractionation results in only a small peak in the plasma concentration of epirubicin leading to low maternal toxicity and only slight placental transfer of the drug [12]. In addition, weekly treatment allows close monitoring of the pregnancy, which reassures both the patient and the treating physician. It also results in shorter nadir periods and thus allows easy interruption of the pregnancy if needed. However, outside pregnancy, this regimen is not routinely used in the adjuvant setting but it is important to note that the treatment period during pregnancy does not comprise the whole adjuvant treatment period and reverting to more standardized regimens can be done after delivery. Differences between anthracycline-based regimens are at best modest in terms of overall survival and are mainly attributed to the dose of anthracycline, which is very well preserved with a weekly epirubicin dose of 35 mg/m^2^.

Very little data are available regarding the safety of other chemotherapeutic agents. Methotrexate is strongly discouraged as it is used for induction of abortion [13]. Taxanes have been described in the literature in around 30 pregnant breast cancer patients [14]. There is no evidence that they increase the risk of pregnancy complications. Thus they remain the second best option in case anthracyclines are contraindicated for any reason. Data on other agents are even scarcer and thus they should not be considered in treating pregnant breast cancer patients.

Data on trastuzumab are limited to 15 pregnant cases with HER2+ breast cancer with a striking high incidence of oligohydramnios reaching up to 50% [15]. This is secondary to the inhibitory effect of trastuzumab on HER2 expressed on the foetal kidney, which is responsible for the amniotic fluid production [16]. Oligohydramnios was mainly observed in patients exposed to the drug for more then one trimester [17]. Thus trastuzumab is strongly discouraged during pregnancy. As for hormonal therapy, treatment with tamoxifen is contraindicated throughout the course of the pregnancy as it is associated with a considerable risk of foetal congenital anomalies [18].

## Gynecological tumours

Cervical and less commonly ovarian cancers have been diagnosed in women during their pregnancy course. Given their anatomical location, their treatment represents a major challenge. Pelvic surgery remains very challenging during pregnancy as the access is impaired and thus an optimum oncological resection is technically very difficult to achieve [19]. Thus, surgery should not be considered outside centres with experience dealing with pregnant cancer patients. Pelvic radiotherapy should be avoided during gestation, as the risk of foetal loss or malformations are significant [8]. Elective abortion should thus be considered during the first trimester, if the clinical situation mandates a prompt treatment.

A recent systematic review has identified 16, 18, and 20 patients treated with chemotherapy for cervical, non-epithelial and epithelial ovarian cancer, respectively [8]. Concomitant cisplatin and radiotherapy was frequently considered in cervical cancer patients, but spontaneous abortion was encountered in all patients exposed to radiotherapy; cases treated with weekly cisplatin alone had a normal pregnancy outcome. For non-epithelial ovarian cancer, 15/18 cases did not show any signs of pregnancy complications. In the remaining three cases, pregnancy complications were successfully managed with no foetal abnormalities documented. The most frequently used regimen was BEP (bleomycin, etoposide, and cisplatin), which is also considered as the gold standard treatment for non-pregnant women. The combination of paclitaxel and carboplatin was also frequently reported in managing epithelial ovarian cancer during pregnancy, with no serious complications reported.

## Lung cancer

The association between lung cancer and pregnancy is very rare; however it is expected to increase because of the increasing rates of cigarette smoking among young women [20]. A recent systematic review of the literature performed by our group has identified 44 pregnant women were diagnosed with lung cancer during pregnancy [21]. The vast majority was diagnosed with adenocarcinoma and had advanced disease. The prognosis was very poor and most of the patients died within 1 year after delivery. Only six patients were treated with chemotherapy during gestation with platinum-based chemotherapy. Preterm delivery occurred in five of them, which was not unexpected due to the poor maternal conditions and high tumour burden associated with these cases.

Several targeted agents were described in managing patients with advanced non-small cell lung cancer [22]. This includes bevacizumab, a monoclonal antibody against vascular endothelial growth factor (VEGF) that acts as a key regulator of angiogenesis, both physiological (e.g. during embryogenesis and skeletal growth) and pathological (e.g. tumour growth) [23,24]. No reports have been described on the use of bevacizumab in pregnant cancer patients. However, given the vital role of angiogenesis in normal development of the foetus, targeting VEGF could result in serious congenital malformations [15,25,26]. Preclinical models using bevacizumab, thalidomide and other VEGF tyrosine kinase inhibitors (TKIs) were associated with serious pregnancy complications [24,27,28].

Thus, bevacizumab should not be considered in treating pregnant patients with advanced lung cancer or any other disease. As for epidermal growth factor receptor inhibitors (EGFRi), no reports have been reported for cetuximab whereas only one case was unintentionally exposed to the TKI erlotinib during the first trimester with normal pregnancy outcome [29].

Patients with gestational lung cancer should be properly counselled regarding their prognosis and the limited treatment options available. If the patient agrees to proceed with the pregnancy, then a platinum-based chemotherapy could be an option after the first trimester. The combination with either paclitaxel or vinorelbine has been used, without apparent foetal toxicity also in pregnant lung cancer patients. [21].

## Lymphoma

Hodgkin lymphoma (HL) is the most common lymphoma diagnosed during pregnancy. We have recently identified around 70 patients who were treated with systemic chemotherapy during pregnancy [30]. The majority was treated with the standard regimen ABVD (doxorubicin, bleomycin, vinblastine and dacarbazine). Those treated following the first trimester had an uneventful pregnancy course and outcome while, as expected, there was a high incidence of congenital anomalies in those who were treated with chemotherapy during the first trimester (6/17). Thus ABVD, at the standard dose, may be considered for pregnant patients diagnosed with HL starting at the second trimester. In one report, the follow-up of 26 foetuses exposed *in utero* to ABVD or MOPP showed normal development up to 18 years of follow-up, without significant late toxicities [31]. As for radiotherapy, it may be considered after a thorough treatment plan with foetal dose calculation for cases in which the disease is limited to the neck region, but these are very rare situations. Data on non-HL (NHL) are more or less similar to those of HL. CHOP was offered to 15 patients with aggressive lymphomas, with normal pregnancy outcomes [30]. Long-term follow-up of a series of 29 Mexican patients treated with a CHOP-like regimen showed normal development of their infants [31]. Thus for pregnant patients with NHL, CHOP can be considered a safe option.

Rituximab is a monoclonal antibody against CD20 and has shown to improve outcomes in indolent and aggressive NHL [32,33]. No preclinical reproductive toxicity models for rituximab are described in the literature. To date, only seven lymphoma patients have been exposed to rituximab during pregnancy; six in combination with chemotherapy and as a single agent to the seventh patient [15]. The latter had relapsing follicular lymphoma and was exposed unintentionally to rituximab during the first trimester. The drug was stopped and the pregnancy was allowed to continue with normal outcome. The remaining six patients had different types of aggressive NHL and treatment with rituximab was initiated during the second trimester. All seven patients had an unremarkable pregnancy course. However, in three out of seven neonates, CD19+ B cells were either undetectable or severely decreased at birth or shortly after [34–36]. The same was observed in another neonate born for a pregnant woman diagnosed with immune thrombocytopenic purpura and treated with rituximab during pregnancy [37]. The condition was reversible in all cases with all B-cell levels returning to normal within 3–6 months. No significant post-natal infections were encountered and subsequent follow-up revealed adequate response to standard immunization in all four children.

Thus, patients with HL or NHL diagnosed during pregnancy should be treated with standard regimens at the same dosages used in the non-pregnant settings. It is important to note that these patients are at high risk of developing haematological toxicity and thus should be adequately monitored as such toxicities could endanger the pregnancy course and predispose to preterm delivery. The use of granulocyte colony stimulating factors (G-CSFs) has been documented in seven pregnant patients with lymphoma or leukaemia, without complications [30]. Thus G-CSF may be also considered during pregnancy if a high risk of neutropenia is forwarded. The same applies for erythropoietin in the management of anaemia, even if blood transfusions remain the preferred choice.

## Leukaemia

Acute leukaemia (AL) is more frequently diagnosed during the childbearing period and has been more frequently described during pregnancy compared to chronic leukaemia. Acute myeloid leukaemia (AML) is the most common AL diagnosed during pregnancy and more than 100 patients treated with chemotherapy have been documented in the literature [30].

In non-pregnant patients, the combination of cytarabine with either idarubicin or daunorubicin is the most commonly used in treatment of AML during the induction phase [38]. Both combinations were reported during pregnancy with worrying results. Out of 32 patients exposed to cytarabine–daunorubicin-based combination following the first trimester, only 15 had normal pregnancy outcome [30]. Around four foetal deaths were reported in addition to several congenital anomalies. Similar observations were encountered in seven patients exposed to idarubicin. The latter is more lipophilic and thus trans-placental transfer is expected to be higher compared to the other anthracyclines. Hence, a better alternative during pregnancy could be doxorubicin, in which data from breast cancer and lymphoma are reassuring. Moreover, data in managing AML outside pregnancy showed comparable efficacy to daunorubicin and idarubicin [38,39]. Several pregnant AML patients were managed with cytarabine in combination with doxorubicin with good foetal outcome [30].

As for acute lymphoblastic leukaemia, similar observations were made regarding the safety of daunorubicin and idarubicin and thus both should be avoided and replaced by doxorubicin.

Acute promyelocytic leukaemia is a subtype of AML and is characterized by the chromosomal translocation *t*(15/17). It is routinely treated with the same chemotherapeutic regimen as is used in managing AML in addition to all trans retinoic acid (ATRA) that targets this specific translocation. Even though preclinical data suggest a high risk of abnormalities associated with using this agent and thus should be avoided early in pregnancy, approximately 15 pregnant patients were reported to have been exposed to the single agent ATRA, mainly during the second and third trimesters, with normal pregnancy outcomes [30]. A further two documented cases were exposed to this drug during the first trimester also with normal outcomes to the pregnancy. Hence we propose that ATRA can be safely used during pregnancy and in cases where other chemotherapy is needed, doxorubicin remains the anthracycline of choice as highlighted earlier.

Chronic myeloid leukaemia has also been described during pregnancy. It is characterized by the presence of BCR/ABL fusion gene, which is the main driving force for the development and maintenance of the leukaemic clone [40]. Targeting this oncogene with imatinib has yielded extraordinary good results leading to cure in a large fraction of chronic myelogenous leukaemia patients [41]. There are more than 200 documented cases of exposure to imatinib during pregnancy. Most of the published reports describe cases in which the patient was inadvertently pregnant during the treatment course. Early exposure resulted in a high incidence of spontaneous abortions and congenital anomalies in up to 30% of reported cases [30], whereas those who started imatinib following the first trimester did not encounter pregnancy-related complications. Thus, patients should be clearly informed of the risks and advised to take contraception while on imatinib. If conception occurred during the treatment course, the drug should be stopped and the patient should be informed on the potential risk of congenital abnormalities associated with this drug. If the patient wishes to keep the pregnancy, imatinib could be substituted by interferon during the first trimester as it does not cross the placental barrier [42]. Imatinib could be resumed later during the second trimester.

## Conclusions

Managing cancer during pregnancy is feasible. Each patient should be properly counselled and informed that the evidence is scarce, but not absent. Respecting the patient’s autonomy is of extreme importance and the physician’s moral judgment should not influence the patient’s decision. If the patient decides to preserve the pregnancy and receive active treatment, then the decision should be discussed within a multidisciplinary meeting involving not only the oncology team, but also the obstetrician and the neonatologist so that a fairly balanced medical decision, acknowledging the potential benefits and risks for both the mother and the foetus, can be achieved.

## Figures and Tables

**Table 1:** General consideration in managing pregnant women diagnosed with cancer

Surgery	Can be performed anytime during the course of pregnancy. Surgeries with high morbidity (e.g. gastrointestinal/lung) remain discouraged as they could compromise the pregnancy course. If urgently needed, abortion should be considered
Radiotherapy	A general rule is to avoid radiotherapy during pregnancy as possible. However, it is possible to be considered for the cervical and mediastinal region with adequate uterine shielding. This should be only performed in centres with experience in managing patients diagnosed with cancer during pregnancy
Chemotherapy	Avoid during the first trimester as it is associated with high risk of congenital anomalies and spontaneous abortion Fractionation of the dose on weekly bases allows easy pregnancy monitoring, easy interruption of chemotherapy if needed. Also results in shorter nadir periods which facilitate easy interruption of pregnancy if needed as well. This approach is highly encouraged
Hormonal agents	Should be avoided all through the pregnancy course
Time of delivery	Avoid delivery in the nadir periods Avoid delivery before week 35 to avoid prematurity-related complications
